# The Association between Absence of Abdominal Pain and Mortality in Lower Intestinal Perforation in Patients with Autoimmune Rheumatic Diseases

**DOI:** 10.1155/2019/5381453

**Published:** 2019-02-17

**Authors:** Yukari Endo, Yoshiyuki Abe, Shingo Kawano, Taiki Ando, Kazuhiro Sakamoto, Naoto Tamura

**Affiliations:** ^1^Department of Internal Medicine and Rheumatology, Juntendo University School of Medicine, Tokyo, Japan; ^2^Department of Coloproctological Surgery, Juntendo University School of Medicine, Tokyo, Japan

## Abstract

**Objective:**

To determine mortality and predictive factors for lower intestinal perforation (LIP) among patients with autoimmune rheumatic diseases.

**Methods:**

This retrospective, single-center, observational study analyzed mortality rates in 31 autoimmune rheumatic disease patients with LIP who were admitted to our hospital from January 2002 to June 2017. The primary outcome was the mortality rate during hospitalization.

**Results:**

The median age at the time of LIP was 61 years, and the survival rate at discharge was 64.5%. Eleven patients died of sepsis during hospitalization. Cox univariable analysis for mortality during hospitalization showed that absence of abdominal pain (hazard ratio (HR) 5.61, 95% confidence interval (CI) 1.38–22.9), higher age (HR 1.06, 95% CI 1.01–1.11), chronic kidney disease (HR 6.89, 95% CI 1.85–25.7), systemic vasculitis (HR 3.95, 95% CI 1.14–13.6), higher blood urea nitrogen (HR 1.02, 95% CI 1.01–1.04), higher serum creatinine (HR 1.41, 95% CI 1.06–1.87), and LIP due to malignancy (HR 14.3, 95% CI 1.95–105.1) significantly increased mortality.

**Conclusion:**

Abdominal pain was absent in 16% of LIP patients with autoimmune rheumatic diseases, and this absence was a poor prognostic factor in this cohort. Moreover, higher age, chronic kidney disease, systemic vasculitis, and LIP due to malignancy were associated with significantly increased mortality. Physicians should be aware of LIP in autoimmune disease patients with higher age, chronic kidney diseases, or systemic vasculitis even if patients reveal mild abdominal symptoms.

## 1. Introduction

Lower intestinal perforation (LIP) is rare, but it is a serious complication with a high mortality rate and often requires emergency surgery. The mortality rate of LIP was 12% to 50% in previous reports [1–4]. In patients with rheumatoid arthritis (RA), gastrointestinal perforations were reported to occur most frequently in the lower gastrointestinal tract [5].

Most patients with autoimmune rheumatic diseases receive glucocorticoids (GCs) as well as combination therapy with immunosuppressive agents. Nonsteroidal anti-inflammatory drugs (NSAIDs) are often administered to RA patients and may result in adverse effects such as gastrointestinal ulcer, abscess development, and perforation [6–12]. Similarly, GC administration has also been associated with LIP [12–14].

Few studies have reported the risk factors for gastrointestinal perforation in autoimmune rheumatic disease patients [14–16]. In this study, we examined predictive factors for LIP and mortality rates among patients with autoimmune rheumatic diseases.

## 2. Patients and Methods

### 2.1. Patients

This retrospective, single-center, observational study determined the mortality rate in 31 autoimmune rheumatic disease patients with LIP who were admitted to our hospital from January 2002 to June 2017. All patients fulfilled the classification criteria for their respective autoimmune rheumatic diseases. Among patients with acute abdomen, 43 were diagnosed with gastrointestinal perforation based on clinical manifestations and radiographic findings. Thirty-one of these 43 patients were enrolled after we excluded patients with upper gastrointestinal perforation or perforation of unknown location. In this study, we defined the lower gastrointestinal tract as the area from the jejunum to the rectum. In recurrent cases, evaluations were performed only for the initial episode. Fifty-eight LIP patients without autoimmune rheumatic disease who were admitted to our hospital from January 2010 to June 2017 were enrolled in this study as the control group. Sixty-four patients without autoimmune rheumatic disease were diagnosed with LIP between the observational period, and we excluded 5 patients with traumatic gastrointestinal injury and 1 patient with ingestion accident of toothpick. This study was approved by the ethical committee of Juntendo University Hospital (No. 334).

### 2.2. Clinical Evaluation and Outcomes

Clinical data, including patient demographics, clinical manifestations, laboratory data, treatments, and outcomes, were obtained from medical records. The absence of abdominal pain was defined as a lack of spontaneous abdominal pain with or without abdominal tenderness. Comorbidities included interstitial lung disease, chronic kidney disease (CKD), and diabetes mellitus. CKD was defined in this study as stage G3–5 disease [17]. The primary outcome was non-disease-specific mortality during the hospitalization period. The datasets are available from the corresponding author on reasonable request.

### 2.3. Statistical Analysis

To compare demographic and disease characteristics between groups, the Mann-Whitney U test was used for nonnormally distributed variables. Categorical variables were compared using Fisher's exact test. The survival rates of each group were compared. Kaplan–Meier curves were plotted and evaluated using the log-rank test. Hazard ratios were calculated using Cox regression hazard models. Data are presented as medians (interquartile range (IQR)). Analyses were performed using SPSS 23.0 software (SPSS, Chicago, IL).* P* < 0.05 was considered to be statistically significant.

## 3. Results

The study population consisted of 9 males and 22 females. The median age at the onset of LIP was 61 years (IQR, 47–71 years). All 31 patients received an abdominal CT scan before the operation. The survival rate during hospitalization for LIP was 64.5%. The 1- and 5-year survival rates were the same at 60.9%. Two patients experienced recurrent LIPs and survived after hospitalization of second LIPs.

The underlying diseases in the entire study population were as follows: RA (16%), systemic lupus erythematosus (SLE) (23%), systemic sclerosis (SSc) (10%), polymyositis/dermatomyositis (PM/DM) (3%), mixed connective tissue disease (MCTD) (10%), systemic vasculitides (32%), polymyalgia rheumatica (PMR) (3%), and adult-onset Still's disease (AOSD) (3%). The median duration of the underlying diseases in this study was 76 months (IQR, 6–224 months). Fifteen patients were diagnosed with colonic diverticula before LIP.  Table 1 shows patient characteristics at the time of LIP and subsequent operative procedures, with comparisons between the survivor and the hospital death groups. All 11 deaths were due to sepsis, which was caused by peritonitis, catheter infections, and postoperative pneumonia in 7, 3, and 1 patients, respectively. Comorbidities included interstitial lung disease (23%), CKD (39%), and diabetes mellitus (32%). There was a significantly higher prevalence of CKD in the hospital death group. Blood tests performed at the time of LIP diagnosis showed higher levels of blood urea nitrogen (BUN) and serum creatinine in the hospital death group. Regarding autoimmune rheumatic disease treatment, there was no difference between the 2 groups in the dose of GCs at autoimmune rheumatic disease diagnosis and at LIPs, or in the percentages of patients receiving immunosuppressive agents or NSAIDs at the time of LIP. Regarding biological agents, only 1 patient was treated with infliximab, and no patients were treated with tocilizumab.

The absence of abdominal pain was significantly higher in the hospital death group. Five patients without abdominal pain complained only of abdominal discomfort or appetite loss, and in these cases free air was found on CT imaging. A 50-year-old man with GPA complained of appetite loss, and LIP was found incidentally on abdominal CT. A 43-year-old man with PAN was undergoing treatment for colonic ulcer, and LIP was incidentally found on abdominal CT being performed for therapeutic evaluation. A 52-year-old woman with SLE was hospitalized due to fever and sepsis, and LIP was identified on diagnostic CT. A 71-year-old woman with MPA complained of nausea, and free air was found on X-ray. She was diagnosed with LIP by abdominal CT. An 83-year-old woman with MPA was hospitalized with bloody stool and was diagnosed with LIP by abdominal CT. As the criteria for abdominal radiological examination were not defined in this study, it was the preference of the physicians whether to do the examination or not. We did not routinely perform abdominal X-ray or CT on all cases and however selectively examined in some cases. Neither the dose of GCs nor the administration of NSAIDs was associated with the presence or absence of abdominal pain.

**Figure 1:**
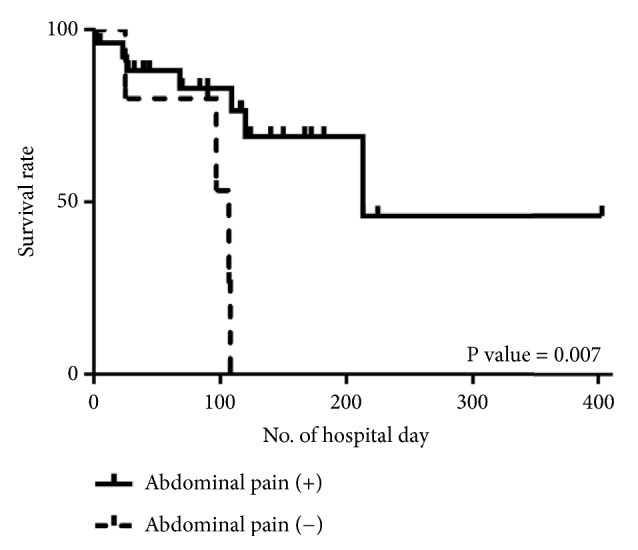
Kaplan–Meier curves for mortality during hospitalization. The log-rank test results in the groups with and without abdominal pain were* P* = 0.007.

Figure 1 shows Kaplan–Meier curves for mortality during hospitalization. The log-rank test results in the groups with and without abdominal pain were* P* = 0.007, respectively.

Causes and locations of LIP were distributed unevenly between the survivor and hospital death groups. We determined that LIPs in 5 cases were due to autoimmune rheumatic diseases: 3 cases in which vasculitis was demonstrated pathologically at the perforated location and 2 cases in which LIP was caused by SSc intestinal lesions. One patient with EGPA had multiple ulcers and LIPs in the ileum, and eosinophil infiltration and fibrinoid necrosis in blood vessels were found around the ulcers. In the patients with PAN or MPA, fibrinoid necrosis was demonstrated in blood vessels around ulcers. In the patient with SSc, intestinal peristalsis was reduced due to intestinal lesions, and LIP occurred due to parasitic ileus. All patients with LIP due to malignant tumors had colorectal cancer. One patient exhibited simultaneous perforation of the colon and appendix, while another had concurrent perforation of the colon and rectum. Fourteen patients received direct hemoperfusion with polymyxin B immobilized fiber (PMX-DHP) after surgery. After LIPs, only one patient with EGPA was treated with intensive immunosuppressive therapy which included intravenous cyclophosphamide pulse therapy. Other patients, including patients who had LIP caused by autoimmune rheumatic disease, were treated without immunosuppressive agents because they were considered that had high risk for critical infection.

In the relationship between mortalities during hospitalization, each variable was evaluated by Cox univariate analysis (Table 2). Higher age (HR 1.06, 95% CI 1.01–1.11,* P* = 0.02), CKD (HR 6.89, 95% CI 1.85–25.7,* P* = 0.004), vasculitis (HR 3.95, 95% CI 1.14–13.6,* P* = 0.03), absence of abdominal pain (HR 5.61, 95% CI 1.38–22.9,* P* = 0.02), higher BUN (HR 1.02, 95% CI 1.01–1.04,* P* = 0.014), higher serum creatinine (HR 1.41, 95% CI 1.06–1.87,* P* =0.02), and LIP due to malignancy (HR 14.3, 95% CI 1.95–105.1,* P* = 0.009) significantly increased mortality.

We compared the LIP patients with and without autoimmune rheumatic disease (Supplemental Table S1). In control group, 11 patients had history of any malignancies, 5 patients had chronic renal failure on hemodialysis, and each 1 patient had ulcerative colitis, Crohn's disease, bronchial asthma, and myasthenia gravis. Crohn's disease patient had LIP due to Crohn's disease. The details of 11 malignancies were the following: lung cancer in 2 patients, testicular cancer in 2, astrocytoma on 1, maxillary sinus carcinoma in 1, breast cancer in 1, uterus cancer in 1, cervix cancer in 1, rectal cancer in 1, and multiple myeloma in 1. Four patients were treated with NSAIDs for lumbar spinal canal stenosis, osteoarthritis, or cancer pain. Since the preservation periods of the medical record were different, the observational periods did not match between the two groups. The patients with autoimmune rheumatic disease group included more females, less body weight, more frequency of interstitial lung disease, greater glucocorticoid dose and immunosuppressant, lower level of hemoglobin, and higher level of serum amylase. The patients with autoimmune rheumatic disease group needed longer days of hospitalization and showed higher mortality.

## 4. Discussion

In this retrospective observational study, we examined 31 autoimmune rheumatic disease patients with LIP. Among the variables at the time of LIP diagnosis, higher age, absence of abdominal pain, CKD, vasculitis, higher BUN, higher serum creatinine levels, and LIP due to malignancy were associated with increased mortality. Higher age is well known as a risk factor for mortality in LIP patients [18]. Further, previous studies showed that CKD, elevated BUN, and serum creatinine levels were associated with mortality in patients with gastrointestinal perforations or sepsis [19, 20]. Since there were 2 cases of recurrent LIP [21], all patients should be monitored for recurrence.

LIP is one cause of acute abdomen, which regardless of etiology is characterized by abdominal pain. The previous study reported that masking effect of GC affected absence of abdominal pain in patients with gastrointestinal perforation who received GC therapy [16]. We could not reveal the cause of absence of abdominal pain in this study. However, we considered that the masking effect of GC was one reason similarly in the previous report. In a previous report, RA patients with LIP who complained of acute abdominal pain were treated with conventional synthetic disease-modifying antirheumatic drugs in 90% of cases, tumor necrosis factor-*α* inhibitors in 60% of cases, and tocilizumab in 27% of cases [9]. Because 16% of patients in this study lacked abdominal pain, it was not always clear when LIP occurred and we therefore could not measure the time from onset to surgery. One reason for the poor prognosis of patients without abdominal pain may be delayed discovery.

LIP is rare in the general population, with an incidence rate of about 0.04 per 1000 persons per year [22]. In several studies of RA patients who were not treated with TCZ, the incidence rates of LIP were 0.15–1.3 per 1000 persons per year [5, 23, 24]. In our hospital, the approximate number of outpatients per year was as follows for each autoimmune rheumatic disease, without duplication: 2100, 1050, 450, 180, 280, 240, 190, and 60 patients with RA, SLE, SSc, PM/DM, MCTD, vasculitis, PMR, and AOSD, respectively. Patients with vasculitis had a higher incidence of LIP in this study, but this assessment may not be accurate because the observation period varied among individuals. In a previous report, 26% of vasculitis patients experienced gastrointestinal involvement, including gastrointestinal perforations [25]. We plan to examine the relationship between systemic vasculitides and LIP in the future.

Moreover, we examined the incidence rate of LIP in RA patients and non-RA patients with NSAIDs. In this study, 5 patients with RA had LIP during the observation period of 15.5 years, and the approximate incidence rate was 0.15 per 1000 persons per year, which was the similar to previous studies [5, 23, 24]. All 5 RA patients were treated with GC, and 3 RA patients were also treated with NSAIDs. Several studies reported the scope of adverse effect of NSAIDs, GCs, or disease-modifying antirheumatic drugs [13, 26–34]. Polypharmacy is common in RA patients, and it was associated with increased risk of acute hospitalization in the previous study [35]. Almost 3300 patients (range: 3143–3340) were treated with NSAIDs daily per year from 2010 to 2017 in our hospital except for the department of rheumatology. Three thousand of 3300 patients without autoimmune rheumatic disease were age-matched population as RA patients, and 4 patients in control group were treated with NSAIDs. Since the approximate incidence rate was 0.18 per 1000 persons per year in non-RA patients with NSAIDs, it was similar to that in RA patients. The previous study reported no significant difference in lower gastrointestinal events between RA and osteoarthritis patients with NSAIDs [36]. The limitations of our study were not longitudinal study, the number of patients was too small, and we evaluated heterogenous cause of perforation as one LIP. Since the risk for the incidence of LIP in RA patients was significantly higher than in the general population, the physicians should be aware of the higher incidence of LIP in RA patients.

In patients with LIP, malignancy significantly increased mortality. Previous studies reported that LIP due to malignancy was associated with worse prognosis than LIP due to diverticulitis or ischemic enterocolitis [37, 38]. The location of LIP was not associated with prognosis. Similarly, the choice of surgical procedure did not significantly impact outcomes, which may be due to the appropriate procedure being chosen in each case. In this retrospective study, colostomy and PMX-DHP were performed more frequently in severe patients and the association of these procedures with mortality was therefore difficult to evaluate [39].

The LIP patients with autoimmune rheumatic disease showed higher mortality in the comparison between the LIP patients with and without autoimmune rheumatic disease. There were several biases in this comparison that the control group was heterogeneous, observational periods were different in two groups, and too small numbers of patients were enrolled. However, we may clarify the approximate tending in LIP patients with autoimmune rheumatic disease to have longer days of hospitalization and higher rate of hospital death. We considered that these were associated with the characteristics that the more the frequencies of interstitial lung disease, the greater the glucocorticoid dose and immunosuppressant in the LIP patients with autoimmune rheumatic disease. More females were included in the LIP patients with autoimmune rheumatic disease group because of characteristics of rheumatic disease, and less body weight and lower level of hemoglobin were associated with including more females.

The major limitation of this study was its retrospective, single-center, observational design. We did not perform Cox multivariate analysis because of the small number of cases. In addition, autoimmune rheumatic diseases were evaluated as a group rather than individually. Despite these drawbacks, the finding that the absence of abdominal pain was a risk factor for mortality is an important contribution.

## 5. Conclusion

Absence of abdominal pain, higher age, CKD, systemic vasculitis, higher BUN, higher serum creatinine levels, and LIP due to malignancy was risk factors for mortality in autoimmune rheumatic disease patients with LIPs. Even though the absence of LIP-associated abdominal pain is rare in the general population, 16% of autoimmune rheumatic disease patients reported no abdominal pain in this study. Physicians should be aware of LIP in autoimmune disease patients with higher age, chronic kidney diseases, or systemic vasculitis even if patients reveal mild abdominal symptoms.

## Figures and Tables

**Table 1:** Patient characteristics at the time of LIP diagnosis and subsequent operative procedures.

		Survivors	Hospital deaths	*P* value
		*n* = 20	*n* = 11	
Age, years, median (IQR)		61 (41–66)	71 (49–83)	0.08
Sex, female (%)		15 (75)	7 (64)	0.68
Body weight, kg, median (IQR)		43 (36–53)	52 (39–70)	0.09
History of abdominal surgery, *n* (%)		3 (15)	4 (36)	0.21
Interstitial lung disease, *n* (%)		2 (10)	5 (45)	0.07
Chronic kidney disease, *n* (%)		4 (20)	8 (73)	0.007*∗∗*
Diabetes mellitus, *n* (%)		6 (30)	4 (36)	1.00
Underlying disease	RA, *n* (%)	4 (20)	1 (9)	0.63
	SLE, *n* (%)	6 (30)	1 (9)	0.37
	SSc. *n* (%)	2 (10)	1 (9)	1.00
	PM/DM, *n* (%)	1 (5)	0 (0)	1.00
	MCTD, *n* (%)	2 (10)	1 (9)	1.00
	Vasculitis, *n* (%)	4 (20)	6 (55)	0.11
	PMR, *n* (%)	0 (0)	1 (9)	0.36
	AOSD, *n* (%)	1 (5)	0 (0)	1.00
Duration of underlying disease, months, median (IQR)		108 (62–76)	26 (61–83)	0.57
Dosage of GCs at autoimmune rheumatic disease diagnosis, mg/day, median (IQR)		25 (11–48)	20 (8–30)	0.26
Dosage of GCs at LIPs, mg/day, median (IQR)		15 (10–40)	30 (15–40)	0.23
Immunosuppressive agents at perforation, *n* (%)		7 (35)	7 (64)	0.26
NSAIDs at the time of perforation, *n* (%)		3 (15)	1 (9)	1.00
Absence of abdominal pain at perforation, *n* (%)		1 (5)	4 (36)	0.042*∗*
White blood cell count, /*μ*l, median (IQR)		9750 (6650–14900)	9400 (5200–14000)	0.67
Lymphocyte count, /*μ*l, median (IQR)		455 (314–723)	418 (194–1190)	0.58
Hemoglobin, g/dL, median (IQR)		9.9 (8.6–11.7)	9.6 (8.5–11.7)	1.00
Albumin, g/dL, median (IQR)		2.5 (2.1–2.9)	2.8 (2.4–3.3)	0.45
Lactate dehydrogenase, IU/L, median (IQR)		219 (171–408)	241 (187–397)	0.43
Blood urea nitrogen, mg/dL, median (IQR)		18 (11–26)	37 (26–74)	0.008*∗∗*
Serum creatinine, mg/dL, median (IQR)		0.59 (0.36–1.02)	1.32 (0.81–3.83)	0.002*∗∗*
Amylase, IU/L, median (IQR)		91 (54–284)	89 (73–368)	0.64
Plasma sodium, mmol/L, median (IQR)		137 (131–141)	135 (131–139)	0.73
Plasma potassium, mmol/L, median (IQR)		3.8 (3.3–4.5)	4.4 (3.6–4.7)	0.24
CRP, mg/dL, median (IQR)		7.7 (2.6–27.3)	7 (3.4–15.3)	0.76
IgG, g/dL, median (IQR)		968 (676–1361)	892 (771–1142)	0.70
Cause of perforation	Diverticulitis, *n* (%)	10 (50)	4 (36)	0.71
	Autoimmune rheumatic diseases, *n* (%)	3 (15)	1 (9)	1.00
	Malignancy, *n* (%)	1 (5)	2 (18)	0.28
	Unknown, *n* (%)	6 (30)	4 (36)	1.00
Perforation location	Ileum, *n* (%)	4 (20)	1 (9)	0.63
	Appendix, *n* (%)	3 (15)	0 (0)	0.54
	Colon, *n* (%)	13 (65)	9 (82)	0.43
	Rectum, *n* (%)	1 (5)	2 (18)	0.28
Elective operation, *n* (%)		1 (5)	0 (0)	1.00
Emergent operations, *n* (%)		19 (95)	11 (100)	1.00
Operations	Colostomy, *n* (%)	6 (30)	6 (55)	0.26
	Hartmann's operation, *n* (%)	8 (40)	4 (36)	1.00
	Resection, *n* (%)	4 (20)	0 (0)	0.27
	Appendectomy, *n* (%)	1 (5)	0 (0)	1.00
	Drainage for perforation and omental flap, *n* (%)	1 (5)	1 (9)	1.00
PMX–DHP, *n* (%)		9 (45)	5 (45)	1.00
Methylprednisolone pulse therapy after LIPs, n (%)		0 (0)	0 (0)	N/A
Dosage of GCs after LIPs, mg/day, median (IQR)		15 (10–30)	20 (10-55)	0.43
Immunosuppressive agents after LIPs, n (%)		1 (5)	0 (0)	0.65
Days in ICU, median (IQR)		7 (2–11)	13 (6–18)	0.15
Days of hospitalization, median (IQR)		103 (41–163)	97 (25–109)	0.21

IQR: interquartile range, RA; rheumatoid arthritis, SLE: systemic lupus erythematosus, SSc: systemic sclerosis, PM/DM: polymyositis/dermatomyositis, MCTD: mixed connective tissue disease, PMR: polymyalgia rheumatica, AOSD: adult-onset Still's disease, GCs; glucocorticoids, LIPs; lower intestinal perforations, NSAIDs: non-steroidal anti-inflammatory drugs, WBC: white blood cell, CRP: C-reactive protein, IgG: immunoglobulin G, PMX-DHP: direct hemoperfusion with polymyxin B–immobilized fiber, ICU: intensive care unit, N/A: not applicable.

*∗P *< 0.05.

*∗∗P *< 0.01.

**Table 2:** Factors associated with mortality during hospitalization in univariate analysis.

		OR	95% CI	*P* value
Age, years		1.06	1.01–1.11	0.02*∗*
Sex, female		0.57	0.17–1.94	0.36
Body weight, kg		1.04	0.99–1.09	0.08
History of abdominal surgery		2.12	0.63–7.10	0.23
Interstitial lung disease		3.15	0.96–10.3	0.06
Chronic kidney disease		6.89	1.85–25.7	0.004*∗∗*
Diabetes mellitus		1.18	0.35–3.92	0.79
Underlying disease	RA	0.65	0.08–5.09	0.68
	SLE	0.24	0.03–1.87	0.17
	SSc	0.55	0.07–4.38	0.57
	PM/DM	0.05	0.0–2914.8	0.58
	MCTD	0.73	0.09–5.89	0.77
	Vasculitis	3.95	1.14–13.6	0.03*∗*
	PMR	9.23	0.96–88.8	0.054
	AOSD	0.05	0.0–1.3*∗*10^23^	0.90
Duration of underlying disease, months		1.00	0.9–1.002	0.19
Dosage of GCs at autoimmune rheumatic disease diagnosis, mg/day		0.98	0.95–1.02	0.28
Dosage of GCs at LIPs, mg/day		1.01	0.98–1.04	0.57
Immunosuppressive agents at perforation		3.05	0.78–11.9	0.11
NSAIDs at the time of perforation		0.82	0.10–6.70	0.85
Absence of abdominal pain at perforation		5.61	1.38–22.9	0.02
White blood cell count, /*μ*l		1.00	0.99–1.01	0.84
Lymphocyte count, /*μ*l		1.00	1.00–1.00	0.73
Hemoglobin, g/dL		1.08	0.82–1.41	0.59
Albumin, g/dL		1.09	0.42–2.81	0.87
Lactate dehydrogenase, IU/L		1.00	1.00–1.00	0.18
Blood urea nitrogen, mg/dL		1.02	1.01–1.04	0.014*∗*
Serum creatinine, mg/dL		1.41	1.06–1.87	0.02*∗*
Amylase, IU/L		1.00	1.00–1.00	0.95
Plasma sodium, mmol/L		0.98	0.87–1.10	0.73
Plasma potassium, mmol/L		1.93	0.75–4.93	0.17
CRP, mg/dL		0.99	0.94–1.04	0.63
IgG, g/dL		1.00	1.00–1.00	0.74
Cause of perforation	Diverticulitis	1.08	0.33–3.55	0.90
	Autoimmune rheumatic diseases	0.40	0.05–3.10	0.38
	Malignancy	14.3	1.95–105.1	0.009*∗∗*
	Unknown	0.96	0.23–2.59	0.67
Perforation location	Ileum	0.52	0.07–4.09	0.53
	Appendix	0.04	0.0–24964	0.64
	Colon	1.79	0.39–8.24	0.46
	Rectum	1.68	0.36–7.84	0.51
Elective operation		0.05	0.0–2914.8	0.58
Emergent operations		22.37	0.0–1.4*∗*10^6^	0.58
Operations	Colostomy	2.26	0.66–7.75	0.19
	Hartmann's operation	0.73	0.21–2.48	0.62
	Resection	0.04	0.0–28.3	0.33
	Appendectomy	0.05	0.0–9.9*∗*10^8^	0.80
	Drainage for perforation and omental flap	5.24	0.54–51.1	0.15
PMX–DHP		0.81	0.25–2.57	0.71
Methylprednisolone pulse therapy after LIPs		N/A		
Dosage of GCs after LIPs, mg/day		1.02	0.99–1.04	0.25
Immunosuppressive agents after LIPs		0.05	0.0–3.9*∗*10^7^	0.77
Days in ICU		1.14	0.99–1.31	0.06

OR: odds ratio, CI: confidence interval, RA: rheumatoid arthritis, SLE: systemic lupus erythematosus, SSc: systemic sclerosis, PM/DM: polymyositis/dermatomyositis, MCTD: mixed connective tissue disease, PMR: polymyalgia rheumatica, AOSD: adult-onset Still's disease, GCs; glucocorticoids, LIPs; lower intestinal perforations, NSAIDs: nonsteroidal anti-inflammatory drugs, WBC: white blood cell, CRP: C-reactive protein, IgG: immunoglobulin G, PMX-DHP: direct hemoperfusion with polymyxin B–immobilized fiber, ICU: intensive care unit, and N/A: not applicable.

*∗P *< 0.05.

*∗∗P *< 0.01.

## Data Availability

The data used to support the findings of this study are available from the corresponding author upon request.

## Supplementary Materials

Supplementary Materials Supplementary Table S1: The comparison of patient characteristics between the patients with and without rheumatic disease.
