# Supplementary material for: The Association between Absence of Abdominal Pain and Mortality in Lower Intestinal Perforation in Patients with Autoimmune Rheumatic Diseases
Source: Biomed Res Int. 2019 Feb 17;2019:5381453. doi: 10.1155/2019/5381453 (PMC6398054; doi:10.1155/2019/5381453)
Supplement: Supplementary Materials — Supplementary Table S1: The comparison of patient characteristics between the patients with and without rheumatic disease. [file 5381453.f1.pdf]

Supplemental table 1. The comparison of patient characteristics between the patients with and without rheumatic disease.

		Autoimmune rheumatic disease group n = 31	Control group n = 58	*P* value
Age, years, median (IQR)		61 (47–71)	69.5 (56–78)	0.08
Sex, female (%)		22 (71)	25 (43)	0.02*
Body weight, kg, median (IQR)		46 (38–56)	56 (47–62)	0.008**
History of abdominal surgery, *n* (%)		7 (23)	17 (29)	0.62
Interstitial lung disease, *n* (%)		7 (23)	0 (0)	<0.001**
Chronic kidney disease, *n* (%)		12 (39)	12 (21)	0.08
Diabetes mellitus, *n* (%)		10 (32)	10 (17)	0.11
Dosage of PSL at diagnosis of CTD, mg/day, median (IQR)		30 (10–40)	0 (0–0)	<0.001**
Dosage of PSL at perforation, mg/day, median (IQR)		19.5 (10.8–40)	0 (0–0)	<0.001**
Immunosuppressive agents at perforation, *n* (%)		14 (45)	1 (2)	<0.001**
NSAIDs at the time of perforation, *n* (%)		4 (13)	4 (7)	0.43
Absence of abdominal pain at perforation, *n* (%)		26 (84)	57 (98)	0.02*
White blood cell count, /µl, median (IQR)		9600 (6600–14000)	8300 (4700–12875)	0.36
Lymphocyte count, /µl, median (IQR)		445 (264–938)	590 (431–1118)	0.08
Haemoglobin, g/dL, median (IQR)		9.7 (8.5–11.7)	12.3 (10.5–14.6)	<0.001**
Albumin, g/dL, median (IQR)		2.5 (2.2–3.0)	2.85 (2.3–3.6)	0.19
Lactate dehydrogenase, IU/L, median (IQR)		234 (176–397)	196 (166.25–274.5)	0.07
Blood urea nitrogen, mg/dL, median (IQR)		20 (14–39)	20 (14–37.25)	0.86
Serum creatinine, mg/dL, median (IQR)		0.74 (0.41–1.46)	0.835 (0.64–1.7)	0.12
Amylase, IU/L, median (IQR)		89 (61–296)	57 (41–96)	0.006**
Plasma sodium, mmol/L, median (IQR)		136 (131–140)	138 (135–141)	0.13
Plasma potassium, mmol/L, median (IQR)		3.9 (3.5–4.5)	4.05 (3.65–4.525)	0.84
CRP, mg/dL, median (IQR)		7.4 (3.4–21.1)	15.25 (3.9–21.1)	0.71
IgG, g/dL, median (IQR)		951 (680–1297)	N/A	N/A
Cause of perforation	Diverticulitis, *n* (%)	14 (45)	22 (38)	0.65
	Autoimmune rheumatic diseases, *n* (%)	4 (13)	1 (2)	0.048*
	Malignancy, *n* (%)	3 (10)	14 (24)	0.16
	Ischemia, *n* (%)	0 (0)	6 (10)	0.09
	Unknown, *n* (%)	10 (32)	15 (26)	0.62
Perforation location	Ileum, *n* (%)	5 (16)	8 (14)	0.76
	Appendix, *n* (%)	3 (10)	5 (9)	1.00

	Colon, *n* (%)	22 (71)	40 (69)	1.00
	Rectum, *n* (%)	3 (10)	4 (7)	0.69
Elective operation, *n* (%)		1 (3)	1 (2)	1.00
Emergent operations, *n* (%)		30 (97)	57 (98)	1.00
Operations	Colostomy, *n* (%)	12 (39)	14 (24)	0.22
	Hartmann's operation, *n* (%)	12 (39)	20 (34)	0.82
	Resection, *n* (%)	4 (13)	16 (28)	0.18
	Appendectomy, *n* (%)	1 (3)	6 (10)	0.41
	Drainage for perforation and omental flap, *n* (%)	2 (6)	1 (2)	0.28
	Simple closure, *n* (%)	0 (0)	2 (3)	0.54
PMX–DHP, *n* (%)		14 (45)	22 (38)	0.65
Methylprednisolone pulse therapy after LIPs, n (%)		0 (0)	0 (0)	N/A
Dosage of GCs after LIPs, mg/day, median (IQR)		15 (10–40)	0 (0–0)	<0.001**
Immunosuppressive agents after LIPs, n (%)		0 (0)	0 (0)	N/A
Days in ICU, median (IQR)		7 (2.8–13.8)	4.5 (2.8–9)	0.22
Days of hospitalization, median (IQR)		97 (39–140)	36.5 (22.8–53)	<0.001**
Dead case, *n* (%)		11 (35)	4 (7)	0.002**
The year of surgery, year, median (range)		2009 (2002–2017)	2013 (2010–2017)	<0.001**

IQR: interquartile, PSL: prednisolone, NSAIDs: non–steroidal anti–inflammatory drugs, WBC: white blood cell, CRP: C–reactive protein, IgG: immunoglobulin G, N/A: not applicable, PMX–DHP: direct hemoperfusion with polymyxin B immobilized fiber, ICU: intensive care unit.

*$P < 0.05$.

**$P < 0.01$.
